# Beyond participation: exploring leisure literacy and social adaptation within a physical literacy-informed perspective in adolescence

**DOI:** 10.3389/fspor.2026.1808605

**Published:** 2026-06-29

**Authors:** Ali Selman Özdemir, Aydın Karaçam, Teodora-Mihaela Iconomescu, Laurențiu-Gabriel Talaghir

**Affiliations:** 1 Faculty of Sports Sciences, İstanbul Topkapı University, Istanbul, Türkiye; 2 Faculty of Sports Sciences, Bandırma Onyedi Eylül University, Bandırma, Türkiye; 3 Faculty of Physical Education and Sports, Dunarea de Jos University of Galati, Galati, Romania

**Keywords:** adolescence, leisure literacy, physical education, physical literacy, social adaptation

## Abstract

Physical literacy (PL) has emerged as a holistic framework supporting lifelong engagement in physical activity and well-being. Although PL research increasingly emphasizes social and contextual dimensions, less is known about how complementary competencies, such as leisure literacy, are associated with adolescents’ social adaptation. This study aimed to examine the relationship between leisure literacy and social adaptation among high school students within a PL-informed and socio-ecological perspective. A cross-sectional relational design was employed with a convenience sample of 386 high school students (aged 14–18 years). Data were collected using the Leisure Literacy Scale and the Social Adaptation Scale. Independent samples *t*-tests and Pearson correlation analyses were conducted. Additional measurement checks were performed for the Social Adaptation Scale in the present high-school sample. The findings indicated a significant positive association between leisure literacy and social adaptation. Higher leisure literacy levels were observed among students who participated regularly in recreational activities, were members of social clubs, reported satisfaction with their leisure experiences, and faced fewer budgetary constraints. Students with higher perceived welfare status also reported higher leisure literacy and social adaptation scores. Because the study was cross-sectional, relied on self-report measures, and did not directly measure PL, the findings should be interpreted as associations within a PL-informed framework rather than as evidence of causal or transfer mechanisms. Overall, the results suggest that leisure literacy is meaningfully associated with adolescents’ social adaptation and may be relevant to educational and recreational contexts that support adolescents’ social participation and holistic development.

## 1 Introduction

Over the past two decades, physical literacy (PL) has become a central framework within physical education, health promotion, and lifelong physical activity research. Rather than reducing development to sport-specific skills or fitness outcomes, PL emphasizes the integration of physical competence, motivation and confidence, knowledge and understanding, and social interaction as foundations for meaningful and sustained engagement in movement (1–3). This holistic orientation has shifted attention toward inclusive, educationally meaningful, and lifelong forms of participation (4).

Contemporary scholarship increasingly conceptualizes PL as a dynamic and context-dependent process shaped by interactions between individuals and their social, pedagogical, cultural, and environmental contexts (2, 5). Schools are therefore critical settings for PL development, particularly during adolescence, when young people experience identity formation, increasing autonomy, and heightened sensitivity to belonging and peer relationships (6, 7). Experiences during this period may influence both future physical activity trajectories and broader patterns of social engagement (8).

Despite this progress, PL research has paid comparatively less attention to how young people understand, value, and organize leisure beyond formal physical education settings (9, 10). Leisure literacy, introduced in leisure and recreation studies, refers to multidimensional capacities related to recognizing the value of leisure, making informed choices, and engaging purposefully in activities that may support personal and social well-being (11–13). Unlike approaches that focus only on participation frequency, leisure literacy foregrounds agency, reflection, and the meaningful use of discretionary time.

Leisure literacy shares conceptual ground with PL through its emphasis on motivation, understanding, and sustained engagement (1, 14), yet it places stronger emphasis on how individuals interpret and organize everyday leisure. For this reason, leisure literacy can be positioned as a potential construct of interest within PL-informed educational perspectives: it may help explain how adolescents make sense of leisure opportunities, apply self-regulation, and engage in activities that support personal and social development (9, 15, 16). This positioning is conceptual rather than causal, particularly because PL was not directly measured in the present study, and therefore requires empirical testing across different developmental and educational settings.

Leisure practices are also socially consequential because they provide opportunities for interaction, cooperation, belonging, and social recognition, which are closely related to social connectedness and adaptation (17–19). Previous research has shown that structured leisure and recreational participation can support social adaptation among young people by promoting peer relationships, participation in shared activities, and adjustment to social environments (20–23). However, the relationship between adolescents' leisure-related competencies and social adaptation remains underexplored. This focus is particularly relevant in secondary school populations because adolescence is widely recognized as a critical period for future health, physical activity trajectories, and social integration (6, 24, 25). From a broader literacy perspective, leisure-related competencies can be understood as contextually embedded practices shaped by social and educational environments (2, 26). Accordingly, the present study examined the association between leisure literacy and social adaptation among high school students within a PL-informed and socio-ecological perspective. In line with contemporary PL scholarship emphasizing holistic development and lifelong engagement (1, 4, 27), it was hypothesized that leisure literacy would be positively associated with social adaptation.

### 1.1 Conceptual framework and theoretical background

#### 1.1.1 Physical literacy as a lifelong, socio-ecological process

Physical literacy (PL) is widely conceptualized as a holistic and lifelong process integrating physical competence, motivation and confidence, knowledge and understanding, and social engagement to support sustained participation in physical activity (1–3). Rather than representing a fixed endpoint, PL is generally understood as a developmental journey shaped by ongoing interactions between individuals and their environments (2, 27).

A socio-ecological perspective emphasizes that PL develops within interacting systems, including pedagogical practices, social relationships, cultural expectations, and institutional opportunities (4, 5). Within this framework, schools and physical education are key contexts for fostering PL during adolescence, when movement-related attitudes, autonomy, and social orientations become increasingly salient (6, 7).

#### 1.1.2 Leisure literacy as a complementary capacity to physical literacy

While PL provides a powerful framework for understanding movement engagement, it does not fully explain how individuals navigate the broader landscape of leisure beyond structured physical education or sport settings. Leisure literacy addresses this broader domain by focusing on individuals' capacity to understand the value of leisure, make informed and autonomous choices, and engage meaningfully in activities that may support well-being and personal development (11–13).

Leisure literacy foregrounds cognitive and reflective dimensions of leisure, emphasizing agency, interpretation, and intentionality rather than participation alone (12). From this standpoint, higher leisure literacy reflects stronger awareness of leisure opportunities, the ability to evaluate them, and a greater orientation toward aligning activities with personal and social needs.

#### 1.1.3 Adolescence, school contexts, and the development of leisure-related competencies

Adolescence represents a critical developmental period for both PL and leisure-related competencies. During secondary school years, young people experience significant physical, cognitive, and social changes, alongside increasing independence in decision-making related to leisure, physical activity, and peer engagement (6, 24). Schools function as key social environments in which adolescents encounter structured opportunities for movement, social interaction, and identity formation (7, 19).

Within this context, pedagogical approaches that support both PL and leisure literacy may help shape adolescents' long-term engagement patterns. Educational experiences emphasizing meaning, autonomy, and inclusion are more likely to foster sustained participation and positive social outcomes than approaches focused only on performance or compliance (4, 9).

#### 1.1.4 Leisure literacy and social adaptation

Beyond individual development, leisure practices are closely linked to social processes. Participation in leisure activities creates opportunities for interaction, cooperation, shared meaning, and social recognition, all of which may support adolescents' adjustment to their social environments (17–19). Social adaptation can be understood as the individual's capacity to participate constructively in social settings, develop a sense of belonging, and adjust to shared norms and expectations within school and community contexts.

In educational settings, social adaptation is reflected in students' ability to establish positive peer relationships, participate in shared activities, and navigate the social expectations of school life (19). Previous research has demonstrated that leisure and recreational participation can facilitate adaptation and integration among young people by promoting peer relationships and collective experiences (20–22). However, participation alone does not guarantee positive social outcomes. The extent to which leisure contributes to social adaptation may depend on individuals' ability to interpret, organize, and sustain their leisure experiences—capacities that are central to leisure literacy (12, 13).

From this perspective, leisure literacy may be understood as a potential social resource that can enhance the adaptive value of leisure participation. Adolescents with higher leisure literacy may be more capable of identifying activities that foster meaningful interaction, navigating social dynamics within leisure settings, and deriving a sense of belonging from shared experiences. Consequently, leisure literacy was expected to be positively associated with social adaptation within school and community contexts.

#### 1.1.5 Conceptual positioning of the present study

Drawing on the above frameworks, the present study positions leisure literacy as a construct examined within a PL-informed, socio-ecological perspective. While PL provides an overarching framework for holistic movement development, leisure literacy is conceptualized here as a leisure-related competence that may be relevant to adolescents' everyday social participation. Because PL was not directly measured, the present study does not test whether leisure literacy extends, mediates, or transfers PL-related competencies.

Within this framework, social adaptation is conceptualized as the key social outcome associated with adolescents' leisure literacy. The present study does not test mediation, transfer, or causal mechanisms; rather, it examines whether leisure literacy and social adaptation are positively associated in a cross-sectional adolescent sample. In doing so, it responds to current calls within PL scholarship to explore complementary literacies, pedagogical implications, and social outcomes that extend beyond physical activity behaviors alone (1, 4, 27).

## 2 Materials and methods

### 2.1 Research design

This study employed a cross-sectional relational design to examine the association between leisure literacy and social adaptation among adolescents. A relational approach was considered appropriate for investigating whether leisure-related competencies correspond with social outcomes within a PL-informed perspective. However, because data were collected at a single time point and PL was not directly measured, the design does not permit causal inference, conclusions about directional effects, or empirical claims regarding the transfer of PL-related competencies.

### 2.2 Participants

The study sample consisted of 386 high school students (246 females, 63.7%; 140 males, 36.3%) aged between 14 and 18 years (*M* = 16.38, SD = 1.08). Participants were recruited using a convenience sampling method from public high schools. The electronic questionnaire was administered through school-based channels to eligible students, with participation dependent on student and guardian consent. School- or class-level identifiers were not retained in the anonymized dataset; therefore, possible clustering of students within schools or classes could not be modeled statistically. This sampling approach was considered appropriate given the exploratory nature of the study and the focus on examining relationships between psychosocial constructs within a natural educational context. A sensitivity-oriented sample size justification indicated that the sample exceeded the minimum required to detect a small-to-medium correlation (*r* = .20) with 80% power at *α* = .05, based on conventional power analysis procedures (28). Nevertheless, the non-probability sampling strategy and the absence of cluster-level information limit the generalizability of the findings beyond the present sample. Informed consent was obtained electronically from all participants and their legal guardians prior to data collection. Participation was voluntary, and participants were informed of their right to withdraw from the study at any time.

### 2.3 Data collection tools

Data were collected using a structured questionnaire consisting of three sections: (1) demographic information and leisure-related characteristics, (2) the Leisure Literacy Scale, and (3) the Social Adaptation Scale.

Leisure literacy was assessed using the Leisure Literacy Scale developed by Arslan (11). The scale consists of 21 items organized into three subdimensions: Fundamental Leisure Literacy, Functional Leisure Literacy, and Practical Leisure Literacy. Items are rated on a five-point Likert scale ranging from 1 (absolutely unimportant) to 5 (absolutely important). Because the response anchors assess the importance attributed to leisure-related knowledge, awareness, and practices, higher total scores reflect a stronger perceived orientation toward leisure literacy rather than an objective performance-based competence score. Previous validation work reported strong internal consistency for the scale, with Cronbach's alpha coefficients exceeding.80 for all subdimensions, and confirmatory factor analysis supported the three-factor structure (*χ*^2^/*df* = 2.63, RMSEA = .07, CFI = .91). In the present adolescent sample, the scale demonstrated high internal reliability (Cronbach's alpha = .93). Given that the original validation was conducted in a Turkish context but not exclusively with high school students, further adolescent-specific validation remains desirable.

Social adaptation was measured using the Social Adaptation Scale developed by Aydoğdu and Gürsoy (29). The original Turkish title of the instrument is “Sosyal Uyum Ölçeği”; in the present manuscript, the construct is referred to as social adaptation rather than social cohesion in order to align the terminology with the scale's intended construct and validation source. The scale consists of 55 items assessing students' perceived adaptation to their social environment, including aspects of belonging, participation, and adjustment to shared social contexts. Responses are provided on a five-point Likert scale ranging from 1 (strongly disagree) to 5 (strongly agree), yielding total scores between 55 and 275, with higher scores indicating stronger perceived social adaptation. The scale was selected because it provides a comprehensive assessment of social adaptation and demonstrated high internal consistency in its original validation study. Although the original validation was conducted with younger secondary-school students, additional measurement checks were conducted in the present high-school sample. The scale demonstrated excellent internal consistency (McDonald's *ω* = .943; Cronbach's *α* = .943). Item-rest correlations were generally acceptable, and deleting individual items did not meaningfully improve reliability. The data were suitable for exploratory factor analysis [KMO = .742; Bartlett's *χ*^2^(1,485) = 16,562.395, *p* < .001]. A forced one-factor solution indicated a dominant general factor explaining 31.0% of the variance, with most items loading above .30. These findings were interpreted as supporting the use of the total score as a global indicator of perceived social adaptation in the present sample, while not constituting a full age-specific validation of the scale.

### 2.4 Data analysis

Data analysis was conducted using SPSS version 25.0 and JASP statistical software. Prior to the main analyses, the dataset was screened for missing responses, invalid values, outliers, and distributional assumptions. Values were checked against the theoretical response ranges of the scales, and the final analytic dataset included 386 complete responses for the variables used in the analyses. Outliers were inspected using descriptive statistics, theoretical score ranges, and standardized values; no responses were excluded from the final analyses. Normality was evaluated through descriptive indicators and distributional checks. Homogeneity of variances was examined using Levene's test prior to independent samples *t*-tests; where homogeneity assumptions were not met, Welch-adjusted interpretation was considered. Additional measurement checks for the Social Adaptation Scale included Cronbach's alpha, McDonald's omega, item-rest correlations, the Kaiser–Meyer–Olkin measure, Bartlett's test of sphericity, and exploratory factor analysis. Descriptive statistics (means, standard deviations, and frequencies) were calculated for all variables. Independent samples *t*-tests were used to examine differences in leisure literacy and social adaptation according to selected demographic and leisure-related characteristics. Associations between leisure literacy and social adaptation were examined using Pearson's correlation coefficient, together with a 95% confidence interval and the coefficient of determination (*r*²). Because multiple comparisons were performed, *p* values were interpreted alongside effect sizes and with caution regarding potential Type I error. Statistical significance was set at *p* < .05.

## 3 Results

Before the main analyses, measurement checks were conducted for the Social Adaptation Scale in the present high-school sample. The scale demonstrated excellent internal consistency (McDonald's *ω* = .943; Cronbach's *α* = .943). Item-rest correlations were generally acceptable, and deleting individual items did not meaningfully improve the reliability coefficients. The data were suitable for exploratory factor analysis, as indicated by the Kaiser–Meyer–Olkin value (KMO = .742) and Bartlett's test of sphericity, *χ*^2^(1,485) = 16,562.395, *p* < .001. A forced one-factor solution indicated a dominant general factor explaining 31.0% of the variance, with most items loading above.30. Therefore, the total score was retained as a global indicator of perceived social adaptation in the present sample.

Table 1 presents the comparison of leisure literacy and social adaptation scores according to students' perceived welfare status. The results indicated statistically significant differences in both leisure literacy and social adaptation scores. Students who reported a higher perceived welfare status had higher mean scores in leisure literacy (*t* = −3.56, *p* < .001, *d* = 0.54) and social adaptation (*t* = −3.99, *p* < .001, *d* = 0.66) compared with those reporting lower welfare status.

**Table 1:** Differences in leisure literacy and social adaptation scores according to students’ perceived welfare status.

Welfare		*n*	*M*	SD	*t*	*p*	*d*
Leisure literacy	Low	44	73.76	9.89	−3.56	<.001	0.54
	High	342	79.60	10.95			
Social adaptation	Low	44	199.85	30.35	−3.99	<.001	0.66
	High	342	217.35	25.80			

As shown in Table 2, a statistically significant difference was observed in leisure literacy scores based on participation in regular recreational activities. Students who reported engaging in regular recreational activities had significantly higher leisure literacy scores than those who did not (*t* = 4.21, *p* < .001, *d* = 0.43).

**Table 2:** Differences in leisure literacy scores according to participation in regular recreational activities.

Activity		*n*	*M*	SD	*t*	*p*	*d*
Leisure literacy	Yes	174	81.45	10.71	4.21	<.001	0.43
	No	212	76.79	10.87			

Table 3 illustrates the comparison of leisure literacy scores based on students’ membership in social clubs or recreational communities. The analysis revealed a statistically significant difference, with students who were members of such clubs reporting higher leisure literacy scores than non-members (*t* = 3.90, *p* < .001, *d* = 0.47).

**Table 3:** Differences in leisure literacy scores according to membership in social clubs or recreational communities.

Membership		*n*	*M*	SD	*t*	*p*	*d*
Leisure literacy	Yes	70	83.10	9.71	3.90	<.001	0.47
	No	316	77.95	11.09			

The results presented in Table 4 indicate statistically significant differences in both leisure literacy and social adaptation scores according to students' reported budgetary challenges related to leisure activities. Students who reported no difficulty in budgeting for leisure activities demonstrated higher leisure literacy scores (*t* = −2.27, *p* = .023, *d* = 0.24) and higher social adaptation scores (*t* = −3.62, *p* < .001, *d* = 0.37) compared to those who experienced budgetary difficulties.

**Table 4:** Differences in leisure literacy and social adaptation scores according to perceived budgetary challenges for leisure activities.

Budget challenges		*n*	*M*	SD	*t*	*p*	*d*
Leisure literacy	Yes	224	77.86	10.88	−2.27	.023	0.24
	No	162	80.44	10.96			
Social adaptation	Yes	224	211.03	27.59	−3.62	<.001	0.37
	No	162	220.83	25.17			

Table 5 shows the comparison of leisure literacy and social adaptation scores based on students' satisfaction with the leisure activities in which they participated. The findings revealed statistically significant differences in both leisure literacy (*t* = 3.08, *p* = .002, *d* = 0.36) and social adaptation scores (*t* = 3.35, *p* < .001, *d* = 0.40), with higher scores observed among students who reported being satisfied with their leisure activities.

**Table 5:** Differences in leisure literacy and social adaptation scores according to satisfaction with leisure activities.

Satisfaction		*n*	*M*	SD	*t*	*p*	*d*
Leisure literacy	Yes	290	79.89	10.95	3.08	.002	0.36
	No	96	75.97	10.70			
Social adaptation	Yes	290	217.70	26.67	3.35	<.001	0.40
	No	96	207.04	27.12			

The Pearson correlation analysis presented in Table 6 revealed a statistically significant positive relationship between leisure literacy and social adaptation scores [*r* = .474, 95% CI (.393, .548), *p* < .001]. The coefficient of determination indicated that leisure literacy and social adaptation shared approximately 22.5% of their variance (*r*² = .225). This finding suggests a moderate association between the two constructs among high school students.

**Table 6:** Correlation between leisure literacy and social adaptation scores.

Variable	Statistic	Leisure literacy	Social adaptation
Leisure literacy	Pearson Correlation	1	.474**
	Sig. (2-tailed)		<.001
	N	386	386
Social adaptation	Pearson Correlation	.474**	1
	Sig. (2-tailed)	<.001	
	N	386	386

For the correlation between leisure literacy and social adaptation, the 95% confidence interval was [.393, .548], and *r*^2^ = .225.

## 4 Discussion

The present study examined the relationship between leisure literacy and social adaptation among high school students within a PL-informed and socio-ecological perspective. The findings provide cross-sectional evidence that adolescents' perceived leisure-related competencies are meaningfully associated with their perceived adaptation to social environments.

One of the central findings of this study is the significant positive association between leisure literacy and social adaptation. This result suggests that adolescents who report a stronger orientation toward understanding, valuing, and organizing leisure also tend to report stronger perceived social adaptation. The moderate correlation and shared variance of approximately 22.5% indicate a meaningful but not exhaustive relationship, suggesting that leisure literacy is one of several factors that may relate to adolescents' social adaptation. While previous research has highlighted the role of leisure and recreational participation in fostering social relationships and adaptation (17, 20–22), the present findings extend this literature by emphasizing leisure literacy—rather than participation alone—as a potentially relevant social resource. This interpretation is consistent with sociological perspectives emphasizing that leisure and sport derive social value from the meanings, relationships, and structures through which participation is organized (30).

From a PL-informed perspective, this finding aligns with conceptualizations that emphasize social engagement and relational dimensions as relevant to holistic development (1, 2). However, because PL itself was not directly measured, leisure literacy should not be interpreted here as an empirically demonstrated mechanism through which PL competencies are transferred into everyday practices. Rather, the findings support the more cautious proposition that leisure literacy may be meaningfully examined alongside PL-informed educational perspectives when considering adolescents' leisure choices and social adaptation. Future longitudinal or model-based studies that directly measure PL are needed to examine these conceptual relationships more directly.

The results further indicate that students who regularly participated in recreational activities and those who were members of social clubs or recreational communities demonstrated higher levels of leisure literacy. These findings are consistent with previous research suggesting that structured leisure environments provide opportunities for experiential learning, social interaction, and the development of leisure-related competencies (12, 13, 20). Within a PL-informed pedagogical framework, structured leisure contexts may be considered important settings beyond school-based physical education because they provide opportunities for social interaction, leisure decision-making, and meaningful participation (4, 9). Importantly, the present findings suggest that it is not merely access to structured activities that matters, but the ways in which adolescents engage with and interpret these experiences—an orientation that is central to leisure literacy.

Students who reported satisfaction with their leisure activities demonstrated higher levels of both leisure literacy and social adaptation. This finding underscores the importance of meaningful engagement in leisure experiences, echoing theoretical perspectives that emphasize autonomy, enjoyment, and personal relevance as key drivers of sustained participation and positive social outcomes (4, 15). From this standpoint, leisure literacy may contribute to adolescents' ability to make choices that align with their interests and needs, thereby potentially enhancing satisfaction and supporting social adjustment. This interpretation is consistent with PL scholarship that highlights the role of motivation and confidence in fostering lifelong engagement in movement and active lifestyles (1, 27).

The observed differences in leisure literacy and social adaptation according to perceived welfare status and budgetary challenges point to the influence of socio-economic conditions on adolescents' leisure experiences. Students who reported fewer financial constraints demonstrated higher levels of leisure literacy and social adaptation, suggesting that access to resources may facilitate opportunities for meaningful and socially supportive leisure engagement. These findings are consistent with socio-ecological perspectives on PL, which recognize that individual competencies are shaped by broader structural and contextual factors, including access to facilities, programs, and supportive environments (5, 8). They also indicate that socioeconomic status, extracurricular participation, and physical activity level may act as confounding variables that simultaneously influence leisure literacy and social adaptation. Because the present analyses were primarily bivariate and group-comparative, these possible confounders should be examined in future multivariable models.

Taken together, the findings of this study support the relevance of examining leisure literacy within PL-informed educational and socio-ecological perspectives. While PL provides a comprehensive framework for understanding movement-related development across the lifespan, leisure literacy offers additional insight into how adolescents may interpret and organize everyday leisure practices that carry social significance. The present study shows a clear association between leisure literacy and social adaptation, but it does not demonstrate causality, mediation, or transfer from PL. In doing so, it contributes to calls within PL research to examine social outcomes, pedagogical implications, and complementary literacies that extend beyond physical activity behaviors alone (4, 27).

## 5 Conclusions

The present study examined the relationship between leisure literacy and social adaptation among high school students within a PL-informed and socio-ecological perspective. The findings indicate that leisure literacy is positively associated with adolescents' perceived social adaptation and is meaningfully related to participation patterns, satisfaction with leisure experiences, and socio-economic conditions.

These results contribute to the growing body of PL-informed scholarship by highlighting leisure literacy as a leisure-related competence that may be relevant to adolescents' everyday social adaptation. While PL provides a holistic foundation for movement engagement across the lifespan, the present study does not directly measure PL and therefore cannot demonstrate that leisure literacy extends or transfers PL-related competencies. Instead, leisure literacy can be cautiously framed as a construct that may help explain how adolescents interpret, organize, and sustain leisure experiences that carry social meaning.

From an educational perspective, the findings suggest the importance of pedagogical approaches that move beyond participation-based objectives toward fostering adolescents' capacity to make informed, meaningful, and autonomous leisure choices. Physical education and school-based programs that explicitly address leisure literacy—by supporting reflection, agency, and value-based engagement—may contribute to adolescents' social adaptation, although intervention studies are needed to confirm this possibility.

At a broader level, the results highlight the need for educational and youth policies that recognize leisure literacy as a transferable life skill with social significance. Ensuring equitable access to structured and meaningful leisure opportunities—particularly for adolescents facing socio-economic constraints—may support the development of leisure literacy and promote social adaptation within school and community settings. These implications should be interpreted cautiously because the present findings are correlational.

## 6 Limitations and future perspectives

Several limitations of this study should be acknowledged. First, the cross-sectional and relational design does not allow for causal inferences regarding the relationship between leisure literacy and social adaptation. Therefore, statements about transfer, mechanisms, or PL-informed implications should be interpreted as conceptual hypotheses rather than empirically demonstrated processes. Longitudinal and intervention-based studies are needed to examine how leisure literacy develops over time and whether it predicts subsequent changes in social outcomes across different life stages.

Second, the use of a convenience sample limits the generalizability of the findings. The results should therefore be interpreted as applicable primarily to the participating students and school context rather than to all adolescents. In addition, because school- and class-level identifiers were not retained, possible clustering of students within schools or classes could not be modeled. Future research should include more diverse and representative samples across different regions, school types, socio-economic backgrounds, and cultural contexts, ideally using multilevel designs when participants are nested within schools or classes.

Third, data were collected using self-report measures, which may be subject to response biases and common method bias because both main variables were assessed through the same method at the same time. In addition, the Leisure Literacy Scale uses importance-based response anchors, and further research should examine its adolescent-specific validity and its relationship with behavioral or performance-based indicators of leisure competence.

Finally, the Social Adaptation Scale was originally developed with younger secondary-school students. Although the present high-school sample demonstrated excellent internal consistency and supplementary measurement checks supported the use of the total score as a global indicator of perceived social adaptation, these checks do not constitute a full age-specific validation of the instrument. Future studies should further examine the factor structure, measurement invariance, and criterion validity of the scale among older adolescents. Because several group comparisons were performed, the possibility of Type I error should also be considered despite the inclusion of effect sizes. Future research aligned with PL scholarship should employ mixed-methods designs, qualitative interviews or observations, regression models, and structural equation modeling to examine how leisure literacy interacts with specific PL domains—such as motivation, confidence, physical activity level, and social relationships—and whether it mediates or moderates the relationship between recreational experiences and social adaptation.

## Data Availability

The raw data supporting the conclusions of this article will be made available by the authors, without undue reservation.
